# Robot-assisted distal gastrectomy for duodenal gastrointestinal stromal tumors adhering to the pancreas: a case report

**DOI:** 10.1093/jscr/rjad024

**Published:** 2023-02-06

**Authors:** Ai Sakai, Jun Kinoshita, Takahisa Yamaguchi, Koichi Okamoto, Hideki Moriyama, Keishi Nakamura, Itasu Ninomiya, Noriyuki Inaki

**Affiliations:** Department of Gastrointestinal Surgery/Breast Surgery, Kanazawa University Graduate School of Medical Sciences, Kanazawa, Ishikawa, Japan; Department of Gastrointestinal Surgery/Breast Surgery, Kanazawa University Graduate School of Medical Sciences, Kanazawa, Ishikawa, Japan; Department of Surgery, Ishikawa Prefectural Central Hospital, Kanazawa, Ishikawa, Japan; Department of Gastrointestinal Surgery/Breast Surgery, Kanazawa University Graduate School of Medical Sciences, Kanazawa, Ishikawa, Japan; Department of Gastrointestinal Surgery/Breast Surgery, Kanazawa University Graduate School of Medical Sciences, Kanazawa, Ishikawa, Japan; Department of Gastrointestinal Surgery/Breast Surgery, Kanazawa University Graduate School of Medical Sciences, Kanazawa, Ishikawa, Japan; Department of Surgery, Fukui Prefectural Hospital, Fukui, Japan; Department of Gastrointestinal Surgery/Breast Surgery, Kanazawa University Graduate School of Medical Sciences, Kanazawa, Ishikawa, Japan

## Abstract

Duodenal gastrointestinal stromal tumors (D-GISTs) are uncommon and account for 3–5% of all GISTs. Currently, no established surgical strategy for D-GISTs exists, which mostly depends on tumor size, relation to the ampulla and invasion of the adjacent organ. We report a case of large D-GIST resected by robotic distal gastrectomy. A 62-year-old woman was diagnosed with a 5-cm D-GIST located at posterior wall of the duodenal bulb. Computed tomography findings indicated possible tumor invasion of the pancreas head. Robot-assisted distal gastrectomy was firstly planned and pancreatoduodenectomy was also considered when the tumor was invading to the pancreas. Although tumor was tightly adherent to the pancreas, it could be dissected from the pancreatic head without capsule damage and resected by robotic distal gastrectomy with no postoperative complication. The large D-GIST tightly adherent to the pancreas could be resected by efficiency of the robotic surgery.

## INTRODUCTION

Duodenal gastrointestinal stromal tumors (D-GISTs) are uncommon tumors that account for 3–5% of all GISTs. Resection without capsule damage is considered an ideal treatment for localized GISTs. The surgical strategy for D-GISTs has long been discussed because of the complex anatomy of the pancreaticoduodenal region in addition to other options ranging from local resection to major procedures. In recent years, minimally invasive surgery (MIS) for removal of D-GISTs has received much attention. Herein, we report a case of large D-GIST which was resected by robot-assisted surgery.

## CASE REPORT

**Figure 1:**
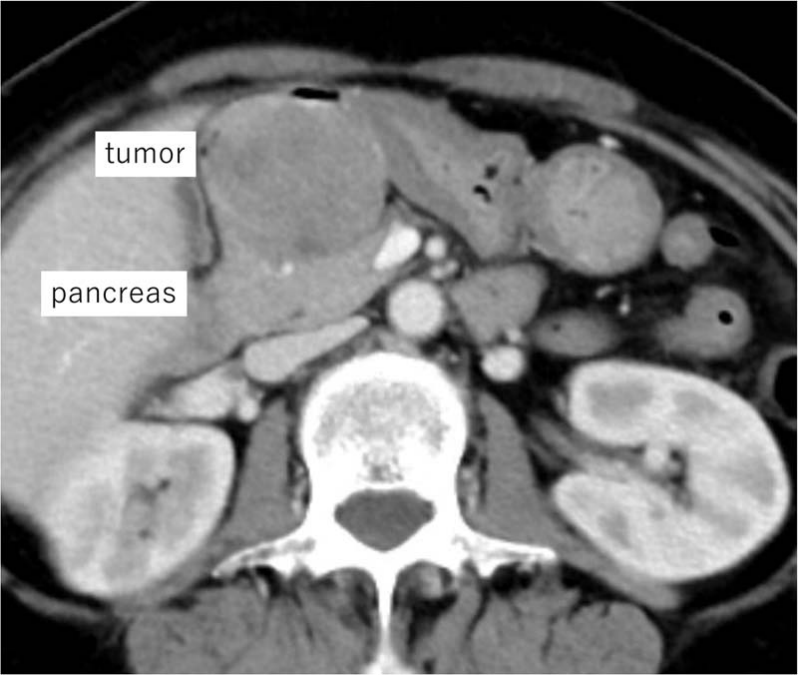
Imaging of the tumor. Contrast-enhanced CT showing a 5 cm large mass with contrast effect on the posterior wall of the duodenal bulb and pancreatic head invasion.

**Figure 2:**
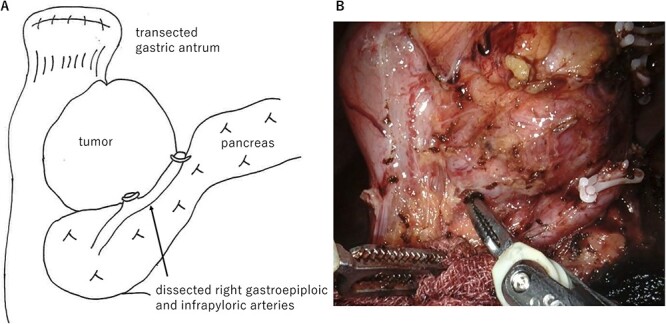
Operative findings. **(A**) Surgical schema. The large tumor was located at the posterior wall of the duodenal bulb and buried in the pancreatic head. Gastric antrum was transected and inverted to the right side. (**B**) Ventral side of the tumor. The prominent part of the pancreatic head was tightly adherent to the tumor. However, the prominent part could be dissected from the tumor by precise manipulation of robotic bipolar Maryland’s forceps.

**Figure 3:**
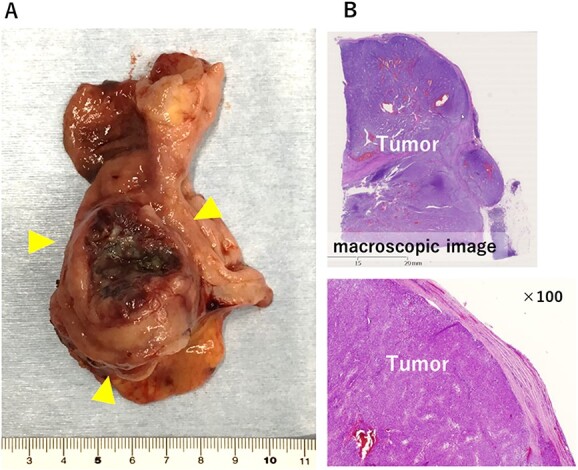
The resected specimen. (**A**) 5.0 × 4.2 × 4.1 cm solid tumor with ulceration was observed in the duodenal bulb. (**B**) Histologically, the tumor capsule was confirmed to be preserved.

A 62-year-old woman presented at our hospital with a primary complaint of orbital pain for the past month. Esophagogastroduodenoscopy showed a duodenal ulcer, whereas a biopsy revealed presence of spindle cells. Immunohistochemistry positively identified c-kit, CD34 and DOG1 and the tumor was diagnosed as a D-GIST. Computed tomography (CT) showed a 5 cm large mass with a contrast effect on the posterior wall of the duodenal bulb and pancreatic head invasion (Fig. 1). Robot-assisted distal gastrectomy was planned. In case the invasion in the pancreatic head could not be detached, an open pancreatoduodenectomy (PD) was to be performed. The operative findings were as follows:

The large tumor was buried in the pancreatic head, which made it difficult to develop the operative field. Gastric antrum was transected and inverted to the right side, which developed the operative field of the dorsal side of the tumor. The prominent part of the pancreatic head was tightly adherent to the tumor. However, the prominent part could be dissected from the tumor without capsular damage by precise manipulation of robotic bipolar Maryland’s forceps (Fig. 2). Finally, the duodenum was transected at the distal part of the tumor and just above the pancreas head with linear stapler. Roux-en Y reconstruction was done, and the stump of duodenum was covered a part of greater omentum by intracorporeal suturing. Operative time was 253 min. Histopathological examination showed proliferation of spindle cells growing in all duodenal layers causing mucosal invasion and ulceration. Although the tumor was tightly adherent to the pancreas, the tumor capsule was preserved and the surgical margins were negative (Fig. 3). No metastasis was observed in the retrieved lymph nodes. The mitotic image showed 100 cells/50 HPF with a high-risk GIST in the Fletcher and Miettinen classification. The patient was discharged without any postoperative complication on the postoperative day 11. The patient received adjuvant therapy with imatinib mesylate, and no recurrence was detected at the 16 month-follow-up.

## DISCUSSION

From the experience of this case, two significant points can be discussed. The first is the surgical strategy for localized D-GISTs. The second is the application of robotic surgery for the large D-GISTs.

Regarding the first issues, various procedures have been reported such as local resection, distal gastrectomy, pancreas-sparing duodenectomy and PD. Although PD may be indicated for complete resection with tumor invasion into the ampulla or pancreas, it should be avoided to reduce operative morbidity and allow function preservation [1]. Several phase II studies have suggested that neoadjuvant imatinib for marginally resectable GISTs reduces tumor size, thereby reducing the risk of tumor rupture and resulting in organ-preserving surgery [2]. However, whether it improves the rate of R0 resection or overall survival is not proven. If neoadjuvant therapy is being considered, it is recommended to confirm tumor mutations via biopsy, although this usually takes several weeks. We considered the dissection from the pancreatic head to be feasible based on the characteristics of GISTs showing expansive growth. Considering the possibility of failure of neoadjuvant imatinib, we decided to precede the surgery.

Recently, several reports indicate that MIS is an effective approach for localized GISTs with favorable outcomes and low rates of morbidity [3]. Lee *et al*. reported that MIS could be utilized for limited resection of D-GISTs with early recovery, although the survival outcomes were insignificantly different between MIS and open surgery [3]. However, conventional laparoscopic surgery has disadvantages, such as limited manipulation of forceps and unnatural positioning of the surgeon, which increase the risk of damage to the capsule. Therefore, the National Comprehensive Cancer Network limits the laparoscopic approach to GISTs <2 cm [4].

The second argument is about the application of the robotic surgery as MIS. The robotic surgery was introduced to overcome the technical limitations of conventional laparoscopy. The advantage to perform procedures with a high operative range by multi-joint forceps of robotic surgery offers an ergonomic resection for the surgeon, which is beneficial in duodenal resection because of its complicated surgical anatomy. For these reasons, the efficacy of robotic surgery for benign and low-grade malignant diseases of the duodenum has been reported. Downs-Canner *et al*. conducted a multicenter cohort study on the outcomes of robotic surgery in 26 cases of benign duodenal tumors [5] and reported acceptable morbidity of greater than or equal to Clavien–Dindo III (15%), despite the complexity of the reconstruction procedure.

On the other hand, there are only six case reports [6–9] and one single-center retrospective study [10] of robot-assisted resection for D-GISTs with sufficient clinical information (Table 1). Vicente *et al*. performed local resection in three patients with D-GISTs and concluded that the robotic approach was safe and feasible with acceptable oncological results [7]. Marano *et al*. treated a 6 cm large D-GIST with duodenal-sparing resection and adjuvant imatinib, and no recurrence was observed during 4 years follow-up [8]. Zhou *et al*. reported surgical outcomes of PD (*n* = 5) and limited resection (*n* = 10) by robotic surgery in patients with D-GIST [10]. Although two of these patients were convert to open surgery, they presented that surgical time and blood loss in robotic surgery were decreased compared with those in the open surgery group. In our case, although the tumor was large and adherent tightly to the pancreas, we were able to achieve R0 resection without pancreatic damage.

**Table 1:** Reported cases of robot-assisted resection for D-GISTs

**Case No.**	**Date**	**Author**	**Age**	**Gender**	**Tumor size(cm)**	**Location**	**Surgery**	**Operation time(min)**	**Surgical margins**	**Fletcher Classification**	**Follow-up duration (months)**
1	2014	Parisi [6]	68 year	F	NS	NS	Pylorus-preserving PD	510	Negative	High	18
2	2016	Vicente [7]	70 year	M	5.5	2nd portion	Enucleation	180	Negative	High	34
3	2016	Vicente [7]	65 year	M	3.5	2nd portion	Enucleation	150	Negative	Low	33
4	2016	Vicente [7]	61 year	F	2.4	1st portion	Enucleation	180	Negative	Low	28
5	2020	Marano [8]	49 year	F	6	2nd–3rd portion	Enucleation	NS	Negative	High	48
6	2021	Madeline [9]	45 year	F	4	2nd portion	Enucleation	NS	Negative	Low	6
7	2022	Our case	62 year	F	5	1st portion	Distal gastrectomy	253	Negative	High	16
8	2021	Zhou [10]	≥50 year: 8 <50 year: 7	M: 11 F: 4	<5 cm:10 ≥5 cm: 5	NS	Enucleation: 10 PD: 5	156 ± 55	Negative: 15	High: 1 Intermediate: 1 Low: 13	NS

F: female, M: male, NS: not specified, PD: pancreatoduodenectomy

In conclusion, the D-GIST in our case was adherent to the prominent part of pancreatic head, which could be completely resected avoiding PD and without perioperative complications.

Although further accumulation of clinical data is needed to verify the safety and oncological outcomes, robotic surgery is considered a promising tool for surgical treatment of D-GISTs.
